# Contributions to variability of clinical measures for use as indicators of udder health status in a clinical protocol

**DOI:** 10.1186/1751-0147-48-15

**Published:** 2006-08-22

**Authors:** C Fossing, M Vaarst, H Houe, C Enevoldsen

**Affiliations:** 1 Department of Animal Health, Welfare and Nutrition, Danish Institute of Agricultural Science, 8830 Tjele, Denmark; 2 Department of Large Animal Sciences, The Royal Veterinary and Agricultural University, 2000 Frederiksberg, Denmark

## Abstract

A cross-sectional observational study with repeated observations was conducted on 16 Danish dairy farms to quantify the influence of observer, parity, time (stage in lactation) and farm on variables routinely selected for inclusion in clinical protocols, thereby to enable a more valid comparison of udder health between different herds. During 12 months, participating herds were visited 5 times by project technicians, who examined 20 cows and scored the selected clinical variables. The estimates of effect on variables were derived from a random regression model procedure. Statistical analyses revealed that, although estimates for occurrence of several the variables, e.g. degree of oedema, varied significantly between observers, the effects on many of these estimates were similar in size. Almost all estimates for occurrences of variables were significantly affected either parity and lactation stage, or by both e.g. udder tissue consistency. Some variables, e.g. mange, had high estimates for the farm component, and others e.g. teat skin quality had a high individual component. Several of the variables, e.g. wounds on warts, had a high residual component indicating that a there still was a major part of the variation in data, which was unexplained. It was concluded that most of the variables were relevant for implementation in herd health management, but that adjustments need to be made to improve reliability.

## Background

Mastitis control is a major part of dairy herd management. Important components hereof are the daily decisions regarding type of treatment, drying off of affected quarters and culling and replacement of cows. Many of these decisions are based on the dialogue and interaction with the local veterinarian. Approximately 40% of Danish dairy farmers have contracted their local veterinarian to visit the farm on a monthly basis [1]. At these contracted visits the veterinarian is expected to provide the farmer with an overview of potential health problems in the herd, to suggest solutions to these problems, and to make recommendations regarding strategies for monitoring and reduction of expected herd-specific health risks. This applicability of such advice relies heavily on the quality of information collected regarding health and disease status on the individual farm.

Information like diagnoses at treatment, somatic cell counts (SCC) and results of bacteriological culturing of milk samples from cows high SCC or clinical mastitis are routinely collected in most herds. Due to farm specific factors like differences in farmers' attitudes to disease [2] and recording efficiency, these data are not sufficient and valid for comparison of herds with respect to udder health. For example, the number of treated mastitis cases will reflect the individual farmer's decisions regarding the number of cows to be treated with antibiotics. The true mastitis incidence may therefore be much higher [3]. Furthermore, Bulk SCCs depend on whether the farmer decides to discard the milk and bacteriological shedding, which may be intermittent [4]. Farmer's records concerning reasons for culling are also very problematic as indicators of health [5].

Studies have been carried out to find additional health measures, that allow the farmers and veterinarians to directly follow the development of udder health in the herd [6,7]. These measures should be linked to potential causal factors of poor milk quality and mastitis problems, and must be relatively easy and inexpensive to apply in operational herd management and the everyday collaboration between farmers and their veterinarians. In addition, it should be possible to communicate these measures between different observers. In farms where registrations are made at regular intervals regarding body condition score and fertility, the cost of such udder health examinations would be minimal.

A Danish pilot study conducted on 4 farms with the aim of developing a clinical protocol for udder examination, indicated a strong relationship between selected clinical udder health measures and milk production values [6], but suggested testing applicability and validity of the findings on a greater sample of herds by testing the effect of observer and time (lactation stage and parity). This study was an investigation of the possible influence of the observer on selected recordings of clinical udder health conditions, and examines the contributions to variability in the these recordings from stage of lactation, parity, cow, and farm.

## Materials and methods

The study, set up as a cross-sectional observational study with 5 repeated observations (visits per farm), was executed from January to December 2000

### Selection of herds

Sixteen Danish dairy herds were selected to represent a broad spectrum of herds within a group of 120 herds enrolled in the project 'Kongeaa Projektet' run by the Danish Dairy Board [8].

The key characteristics of the participating herds are presented in table 1.

**Table 1:** Key characteristics of the 16 participating dairy herds, December 2000.

**Herd ID:**	Breed distribution %	**No. Cows per year **	**Type of housing**	**Summer grazing**	**% cows 1. lact**	**% cows 2. lact**	**% cows ≥ 3. lact**	**Calvings per year**	**Cullings per 100 cow years**
1	87^1^	51	Tied	Yes	28.8	36.5	34.6	61	35
2	100^1^	56	Tied	Yes	31.3	33.7	34.9	50	53
3	100^1^	66	Loose	Yes	45.6	25.3	29.1	66	44
4	98^1^	74	Tied	Yes	30.5	34.5	34.8	60	32
5	99^3^	72	Tied	Yes	28.8	35.6	35.4	88	33
6	100^1^	74	Tied	Yes	30.9	33.5	35.4	86	31
7	67^1^	95	Loose	No	28.3	33.3	38.3	107	36
8	100^1^	113	Tied	No	29.1	36.1	34.6	139	43
9	98^1^	102	Loose	Yes	32.2	35.0	32.7	133	52
10	97^1^	70	Tied	No	27.7	33.0	39.2	90	46
11	98^1^	57	Tied	Yes	31.6	32.9	35.3	58	42
12	99^1^	190	Loose	Yes	29.6	34.0	36.3	201	28
13	100^1^	63	Tied	Yes	28.9	34.0	37.0	72	29
14	100^2^	51	Tied	Yes	29.5	33.1	37.3	62	57
15	98^1^	131	Loose	Yes	28.9	33.8	37.2	139	45
16	100^1^	119	Loose	Yes	29.1	34.8	36.0	123	41

1 = Danish Holstein Friesian Breed, 2 = Red Danish Dairy Breed, 3 = Jersey Breed

### Selection of cows

The selected cows were random samples of the lactating cows in the participating herds. In the loose housing systems, the examined cows were positioned at pre-selected places (e.g. second and fifth cow on the left side) in milking parlours. In the tie-stall systems, the examined cows were positioned as every third or fourth cow from a randomly pre-selected starting point in the stable (e.g. fifth cow from the door).

### Clinical examinations

The selected cows were examined by means of visual inspection and palpation of the udder immediately after one of the twice-daily routine milkings.

All examinations were carried out by project technicians experienced with this type of examination, 2 of whom had previously participated in a similar study. In order to calibrate measurements, 2 joint training sessions were organized for all observers before the commencement of the study period, and clinical data collection forms had illustrations of teat and udder shapes printed on the reverse, together with details describing the individual variable categories.

In table 2 the clinical variables are described in more detail.

**Table 2:** Explanation of the scales used in relation to each of the applied clinical variables.

***Variable***	***Explanation of Variable***
**COW LEVEL**	
Soiling – Hind part and tail	1 = slightly dirty, 2 = moderate and obviously dirty, and 3 = Smeared in thick layer of manure.
Udder	
Teats	
Callus, lateral hocks	1 = hairless and slightly hyperkeratosis, 2 = swollen – unable to feel bones, 3 = large as football
Long claws	1 = slight, 2 = obvious and 3 = extreme
Mange	1 = slight, 2 = obvious, 3 = extreme

**UDDER LEVEL**	
Shape	Coded 1–6 : 1 = normal, 2 = long, 3 = withdrawn between hind legs, 4 = asymmetric front/hind, 5 = small and 6 = deep (Rosenberger, 1979)
Asymmetry – Left/Right	Write smallest quarter code if asymmetry present add degree:
Separate variable for front and hind quarters	1 = slight, 2 = obvious, 3 = extreme
Udder tissue consistency	1 = soft, 3 = firm and 5 = hard (½ values allowed)
Oedema	0 = slight, 1 = large areas, 2 = Most of the udder
Signs of clinical mastitis	0 = not present, 1 = present
Distinct nodes palpated in tissue	Write quarter code if present
Nodular tissue – diffuse	Write quarter code if present

**TEAT LEVEL**	
Shape	Coded 1–5 : 1 = normal, 2 = conical, 3 = short, 4 = fleshy, 5 = other (Rosenberger, 1979)
Skin quality	1 = silk, 2 = very smooth, 3 = smooth, 4 = slightly rough, 5 = very rough (½ values allowed)
Wounds on teats	0 = not present, 1 = present
Warts	0 = not present, 1 = <8 and 2 = >8 on all 4 teats together
Wounds on warts	0 = not present, 1 = present
Warts on teat end	1 = warts on teat end, 2 = warts with wound on teat end
Teat end callus	1 = slight, 3 = immediate visible, 5 = extreme (1/2 values allowed)
Scar tissue in teat canal	Write quarter code if present
Oedema/Hard teat	Write quarter code if present

### Data editing and statistical analyses

Each udder variable was analysed using *Proc Mixed *in the SAS Analysis System (Proc Mixed, SAS,1999). The following base-line model was applied:

Outcome_ijk _= β_0 _+ γ_0k _+ μ_0jk _+ β_1jk_DIM_ijk _+ β_2_OBS_jk _+ β_3_PAR_jk _+ β_4_DIM_ijk_*OBS_jk _+ β_5_PAR_jk_*DIM_ijk _+ β_6_OBS_jk_*PAR_jk _+ β_7_DIM_ijk_*OBS_jk_*PAR_jk _+ β_8jk_DIM_ijk_^2 ^+ β_9jk_DIM_ijk_^3 ^+ β_10jk_DIM_ijk_^4 ^+ ε_oijk_

Where Outcome_ijk _is the response (e.g.) of the i-th DIM for the j-th cow in the k-th herd. β_0 _represents average (expected) response, say clinical score, at time = 0 (fixed effect or the intercept).

γ_0k _represents the departure of the k-th herd from the overall mean response (β_0_). That is, the distribution of herd-effects. This (random) variable allows each herd to have a distinct departure from the average response at Time = 0; a so-called herd-effect. It is assumed to be normally distributed with zero mean.

μ_0jk _represents the departure of the j-th cow from the mean response (β_0_) within herd. That is, the distribution of cow-effects. This (random) variable allows each cow to have a distinct departure from the average herd-level response at Time = 0; a so-called cow-effect. It is assumed to be normally distributed with zero mean.

β_1jk_DIM_ijk _represents average (expected) change in response associated with each unit of change in DIM. This is the regression coefficient or fixed effect of DIM (the average slope).

β_2_OBS_jk _represents the average (fixed) effect of observer (if defined with two levels, otherwise the number of coefficients will be equal to the number of levels minus one). That is, an estimate of the difference between observers at DIM = 0.

β_3_PAR_jk _represents the average (fixed) effect of parity (if defined with two levels, otherwise the number of coefficients will be equal to the number of levels minus one). That is, an estimate of the difference between parities at DIM = 0.

The various crossed effects (DIM*OBS, PAR*DIM, etc.) represent the average (fixed) effects of the interactions between the fixed effects

ε_oijk _represents the residual variance of the individual measurements. That is, an estimate of the random variability associated with the individual measurements, when the fixed effects and random (cow) effects were accounted for. This (random) variable is also assumed to be normal distributed with zero mean.

In case of binary response variables a logistic regression model was used. In that case the residual term was binomially distributed. This model operates with the same baseline as the random regression model.

The general modelling strategy was to specify the most complicated model initially and subsequently eliminate statistically non-significant terms. Statistical significance was judged by calculating the difference in -2LogLikelihod values of models using the maximum likelihood function (ML) with and without the factor. Under the null-hypothesis of no effect of the eliminated term this difference follows a chi-square distribution with degrees of freedom equal to the difference in number of parameters in the contrasted models. This test is a so-called likelihood ratio test.

Those variables, which had very few observations in the categories or for which the distribution of the residuals were not normally distributed, were re-grouped to become binary variables and analysed with the Glimmix macro. The transformed variables were: soiling teats (none vs. slight/more), claw length (optimal vs. slight/more over grown, oedema (none vs. present) and warts (less than eight warts on all four teats vs. eight or more warts on all four teats). Additionally udder and teat shape recordings were transformed into dummy variables.

The dichotomous (present vs. not present) outcomes limit recording within animal, therefore the cow component cannot be estimated for these variables if there is no effect of lactation stage. Thus, the estimates from these models must thus be interpreted as results in a cross-sectional study i.e. a chance of observing a given characteristic in an observed cow.

The variance components of farm and individual cow were calculated by the latent variable approach described by Dohoo [9].

## Results

The results of the type 3 F-tests and the analyses of the variance components of farm and individual cow are presented in table 3. This table illustrates the significant terms remaining after reduction of the full model.

**Table 3:** Presentation of the results from type3 F tests and the analyses of the variance components of the herd and cow components of the clinical parameters (obs= Observer, dim=Days in Milk and par=parity).

	**Fixed Effect**							**Random effects**		
			
*Parameter*	*obs*	*dim*	*par*	obs*dim	dim*par	obs*par	*dim*^*n*^	*Farm*	*Cow*	*Residual*
*Soiling legs*	0.003	-	0.002	NS	NS	NS	dim^4 ^P = 0.002	14.0	21.7	64.3
*Soiling udder*	NS	-	NS	NS	NS	NS	dim^4 ^P < 0.001	14.0	8.3	77.7
*Soiling teats*	0.021	NS	NS	NS	NS	NS	NS	25.9		74.1
*Calluses hocks*	-	-	-	0.009	0.006	NS	NS	24.5	25.8	49.7
*Claw length*	0.001	NS	0.001	NS	NS	NS	NS	21.3		78.7
*Mange*	***	NS	0.018	NS	NS	NS	NS	70.0		30.0
*Long udder*	0.003	<0.001	NS	NS	NS	NS	NS	5.0		95.0
*Withdrawn*	N/C	0.066	0.008	N/C	0.016	N/C	NS	34.0		66.0
*Asymmetric*	NS	NS	0.005	NS	NS	NS	NS	10.9		89.1
*Small udder*	N/C	NS	***	N/C	NS	N/C	NS	40.1		59.9
*Deep Udder*	NS	0.044	***	NS	NS	NS	NS	35.0		65.0
*Asym. Fore*	NS	-	-	NS	0.003	NS	NS	5.9		94.1
*Asym. Hind*	NS	NS	NS	NS	NS	NS	NS	28.3		71.7
*Udder consist.*	-	-	-	0.043	NS	0.005	dim^6 ^P < 0.001	2.6	19.4	78.0
*Oedema*	-	***	NS	NS	NS	NS	dim^3 ^P = 0.001	24.4		75.6
*Inflammation*	N/C	0.009	NS	N/C	NS	N/C	NS	9.3		90.7
*Uddernode*	N/C	0.007	0.009	N/C	NS	N/C	NS	89.4		10.6
*Nodular tissue*	N/C	NS	NS	N/C	NS	N/C	NS	53.1		46.9
*Conical teat*	N/C	N/C	N/C	N/C	N/C	N/C	N/C	38.3		61.7
*Short teat*	N/C	0.014	***	N/C	NS	N/C	NS	24.1		75.9
*Fleshy teat*	N/C	NS	N/C	N/C	N/C	N/C	NS	64.1		35.9
*Other/long teat*	N/C	NS	0.017	N/C	NS	N/C	NS	79.4		20.6
*Teatskin*	***	-	NS	NS	NS	NS	dim^6^P = 0.003	7.2	21.1	71.7
*Wounds*	-	-	0.020	0.025	NS	NS	NS	26.0		74.0
*Warts*	NS	-	NS	NS	NS	NS	dim^2 ^P = 0.009	19.6		80.4
*Wounds warts*	***	NS	NS	NS	NS	NS	NS	5.1		94.9
*Warts -tip*	***	NS	NS	NS	NS	NS	NS	15.3		84.7
*Teatend callus*	-	-	-	NS	NS	0.020	dim^6 ^P = 0.019	7.4	44.3	48.3
*Scar*	N/C	NS	N/C	N/C	N/C	N/C	NS	71.1		28.9
*Hardness*	N/C	NS	NS	N/C	NS	N/C	NS	46.7		53.3

Obs = observer

Dim = days in milk

Par = parity

*** P < 0.001

'- 'signifies significant interaction with other parameter, value not relevant

N/C – non convergence of model

NS – not significant

DIM^n ^indicates that this is the last of the polynomials of DIM to be significant, all polynomials up to this link are included in the final model.

As appears from table 3, several of the models did not converge with observer included in the model. This was due to too few observations in the individual categories. However, of those that did, observer effect was significant for the prevalence of 13 of the variables. Lactation stage and parity had a significant effect on the prevalence of 16 and 15 of the analysed 30 udder health measurements, respectively.

In the following section, the size of effect of observer, lactation stage, parity, farm and cow on the estimates of the significantly affected variables will be presented.

### Differences between observers

**Figure 1:**
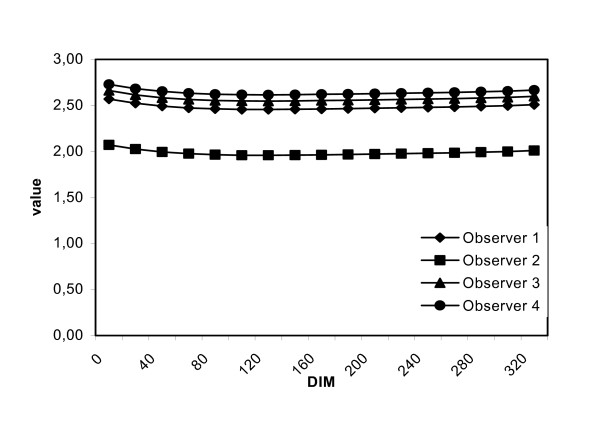
Observer differences in observations of teat skin quality.

The observers made statistically significantly different observations regarding frequency of overgrown claws (estimates varied between 40–60%), chorioptic mange (estimates varied between 0–80%), oedema (the estimate for one observer was 70% and around 30% for the other 3 observers at beginning of the lactation, and 0% for all 4 observers at the end of the lactation), degree of teat skin quality (the estimates for one observer were 0,5 points below the estimates for the other 3), long udder (estimates varied from 0–50%), occurrence of wounds on teats and on warts (estimates varied from 5–30% for both) and occurrence of warts on teat end (estimates varied from 10–36%). For other variables i.e. soiled hind legs (the estimates were 0,5 scoring points below the estimates of the other observers) and teats (estimates varied from 5–9%), hock callus (estimates varied from 0,5–1,3), udder consistency (estimates varied less than 0,2 points for the observers), and teat end callus (estimates varied 0,2 points between observers), predicted estimates did not seem to vary greatly between observers, although the statistical analysis revealed significant differences. An example of the observable magnitude of these differences is demonstrated in figure 1.

It can be seen in figure 1, that the lines corresponding to the individual observer follow a parallel pattern. This indicates that the observers found the same development in relation to parity regarding the variable in question.

### Effect of lactation stage, with no effect of parity

The degree of soiling of the udder decreased 0.25 score values respectively, over the course of lactation. Long udder shape was the only udder shape to be affected by lactation stage and not parity. The prevalence of cows with this udder shape fell during the course of lactation from 50% to 0%. Likewise, the prevalence of cows with udder oedema or udder inflammation fell to near 0% for both variables, though the prevalence fell more sharply for oedema. Although the effect of lactation stage on teat skin quality was significant, the change in the estimate for the score value was very small (figure 1).

**Figure 2:**
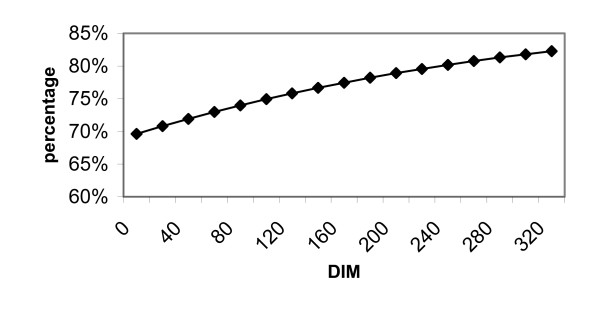
The association between lactation stage (DIM = days in milk) and the occurrence of warts on teats.

Figure 2 illustrates the predicted prevalence of cows with warts on the teats over the course of the lactation. The prevalence of cows with warts on the teats was generally high, and increased to 80% over the lactation, as demonstrated in figure 2.

### Effect of parity with no effect of lactation stage

A higher percentage of older cows were seen to have overgrown claws and mange compared to 1^st ^lactation cows. Regarding the effect of parity on the expected prevalence of the udder shapes, the 'goat' udder was more prevalent in older cows, whereas the small udder shape was much more likely for young cows (24% vs. less than 5% for older cows). Cows with 'other teat shape' (mostly long) were more likely to be second or higher parity cows though there was little difference in the effect on the estimates.

### Effect of lactation stage and parity in combination

Soiling of hind quarters was affected by both lactation stage and parity and decreased approximately 0.5 score values (the predicted score varied between observers) parallel for the three parity groups (no interaction). The percentage of cows with udders between hind legs (withdrawn udders) decreased for second and higher parity cows, but increased for first parity cows.

**Figure 3:**
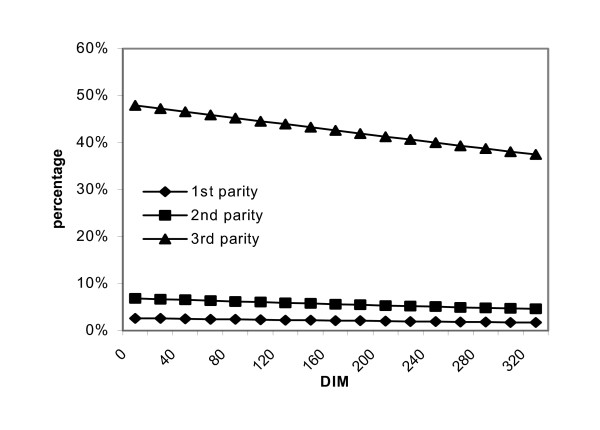
Associations between lactation stage (DIM = days in milk) and deep udder shape.

Figure 3 illustrates the predicted prevalence of cows with deep udder shape over the course of the lactation.

**Figure 4:**
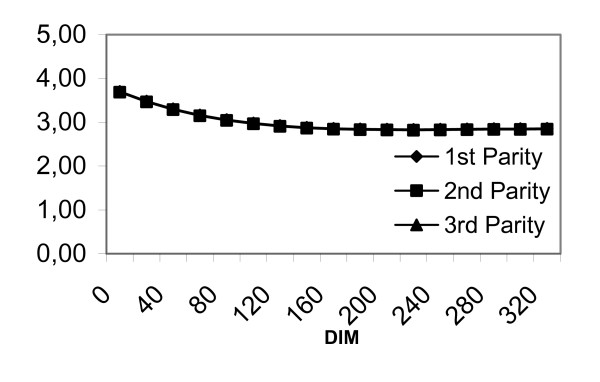
Associations between lactation stage (DIM = days in milk) and predicted degree of udder tissue consistency.

As shown in figure 4 the occurrence of deep udder shape remained constant for first and second parity cows but decreased for third or higher parity cows during the course of the lactation. There was also interaction between lactation stage and parity in relation to occurrence of asymmetric front quarters in that this decreased over the lactation for third or higher parity cows, but increased for first and second parity cows.

Figure 4 illustrates the predicted udder tissue consistency as recorded by one observer over the course of the lactation. As shown in figure 4, the udder consistency decreased over lactation from a score value of 4 to 3 for all 3 parity groups. The estimates for all 3 parities are so similar that only one line may be discerned on the figure.

The likelihood of finding third or higher parity cows with nodes was higher than for the younger cows and increased over the course of lactation; the effect on estimate was small (less than 1% difference). The only teat shape, which was significantly affected by both lactation stage and parity was short teat shape, in that the prevalence of first parity cows with short teats fell from 15% to 5% during lactation and the prevalence of older cows with short teats remained low throughout the lactation period for the other two parity groups.

**Figure 5:**
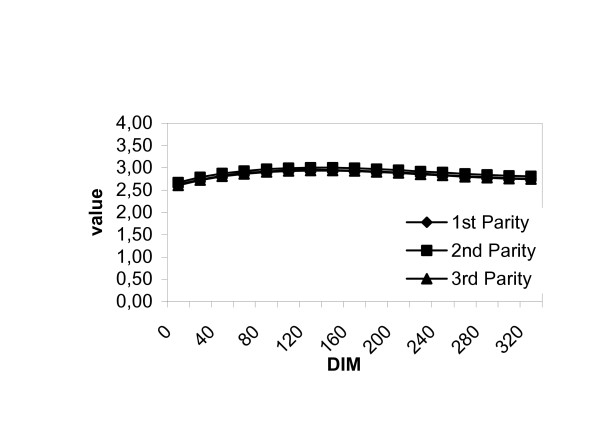
Associations between lactation stage (DIM = days in milk) and predicted degree of teat end callosity.

Figure 5 illustrates the predicted score for grade of teat end callosity for one observer. The estimates for all three parities are so similar that only one line may be discerned on the figure. Figure 5 illustrates that the degree of teat end callus rose slightly during the first weeks of lactation but then decreased until the end of lactation.

### Effects of farm and individual cow

Presence of mange, distinct palpable nodes in the udder, nodular tissue, long and short teats and scar tissue in the teat canal were highly affected by farm. In contrast to this soiling of hind part, udder consistency, teat skin quality and teat canal extraction were affected more by individual cow effects than by farm effect. The only variable to have an equal farm/animal component was callus size.

### Effect of residual

The residual value was high for some variables, indicating that the major part of the variation between observations remained unexplained. These variables were asymmetry of the udder (front and hind quarters), long and 'goat' udders, signs of clinical mastitis and wounds on warts.

## Discussion

### Differences between observers

The observers examined cows in different herds. The possibility that there are systematic differences between herds cannot be completely excluded despite herds being randomly allocated to the observers. However, it was assumed that the variation between cows and herds examined by the same observer was not significantly different from the variation between herds examined by different observers. The seasonal effect on measurement was not covered as a separate part of the analysis and is therefore included in the observer effect.

The results indicate that differences between observers were not eliminated when dealing with variables like soiling of teats, wounds on teats, teat skin quality or udder consistency despite training. This is this unexpected since all observers had had joint training sessions, and the chosen variables express things in a relatively clear way.

Soiling of teats and wounds on teats are examples of differences between observers, where different observers do not follow same pattern. The animals were examined immediately after milking, and therefore, soiling of the teats should not be expected. Since soiling of teats is critical, especially when found immediately after milking, some observers may have been more critical to this and noted very tiny specks of dirt, whereas others have not. In discussions among the observers after the study, disagreement regarding the judgement of necrosis vs. wounds was revealed. This may have been the reason for the recorded differences. More strict definitions and photo references with the categories indicated may be helpful in the classification of variables like soiling and wounds. Neijenhuis et al. [10] found good repeatability when examining for teat end calluses by using a photo guide, and the clinical measurements, which were guided by illustrations on the data sheet, had no significant observer differences. The question may be posed whether or not there may be some individual adjustment of the registrations of the observer to a personal 'norm' over time and whether a photo guide could, for visual measurements, correct for such an adjustment.

For some of the variables e.g. teat skin quality and udder consistency observers did see similar patterns in prevalence of variables, although there were differences in values. Observer variation has previously been described regarding teat-skin quality assessment. For example Rasmussen and co-authors [11] showed that the observer's own skin quality affects the judgement of teat skin quality of the cow. This may have been the case in this study as one of the participants was male. During training sessions, observers had extensive discussions regarding categorisation of udder consistency, and this seems to be a very difficult variable to estimate precisely in a clinical examination. This was reflected in the results. In a variable like this, photos will not help, and descriptions are difficult. In Houe et al [6], it was shown that the best agreement between observers was seen in pathological findings, in contrast to estimating e.g. udder tissue consistency. Generally, observers are more trained in classifying pathological conditions, and a variable like udder tissue consistency is more vague and not obviously connected to pathology. Training of observers at the same time seems to be the only solution in order to improve agreement between observers regarding these types of variables.

### Effect of lactation stage

Soiling of legs and udder was found to be affected by lactation stage, whereas soiling od teats was not. This seems plausible. Cows are often transferred from a clean calving box to the milking stable where the environment may be more contaminated with faeces. Additionally, early in lactation cows may be fed a higher percentage of concentrate to meet high energy demands and this causes faeces to become less viscous [13]. In contrast, soiling of teats may be more influenced by the teat cleaning routine at milking and therefore not significantly influenced by lactation stage.

The udder shapes defined as 'long' and withdrawn udder were all affected by lactation stage. The reason for the decrease seen in the prevalence of these udder shapes during the course of the lactation could very well be that the udder becomes less swollen during the course of the lactation period and therefore relaxes to drop further down. It is well documented that the high levels of cortisol measured immediately after calving often induce oedema [14] and as the levels fall, one would expect the oedema to diminish, as also seen in this study. Similarly, the effect of lactation stage on udder consistency is very plausible. Although the cows were examined immediately after milking, the udder tissue will be more active producing milk at the beginning of the lactation and milk will be stored in ducts and cisterns. Thus, the udder will feel harder on palpation. Thus, the steep rise seen in the prevalence of cows with udder nodes, particularly with respect to the older cows, is plausible. As oedema and udder consistency falls over time, any nodes present in the udder tissue will become easier to feel. In addition, udder nodes are very often a remnant in the form of fibrous tissue from a previous episode of mastitis and, as udder inflammation is also shown by the study to dependent on lactation stage one would expect nodes to be so also.

The results of the study indicated that prevalence of cows with inflammation of the udder decreases over the course of the lactation period. This effect of lactation stage on the occurrence of udder inflammation is well documented. At the start of lactation udder infections have been found to be present at significantly higher levels than in mid- and late lactation [15].

The occurrence of warts on teats rises significantly during the lactation. No evidence based on clinical examinations of cows exists to document that warts on teats should spread between cows in the milking stable. Bovine papilloma, which may cause warts on teats, is known as very contagious, and the results of this study can be viewed as a quantification of this contagiousness.

### Effect of parity

There is a clear increase in the prevalence of older cows with mange infestation. This is an indication of spread of the parasite after introduction to the milking stable. Animals do not seem to rid themselves of this infestation once infested, and as these infested older cows are reintroduced after calving, as they often are, the uninfested first lactation cows pick up the infestation after introduction to the milking herd. The results thus indicate that the prevalence of infested animals will often be linked to the make up of the herd regarding parity distribution.

The fact that cows with asymmetric udders (front vs. hind quarters) are more likely to be third parity or older cows is not surprising. Often this type of asymmetry is caused by the wear of the milking machine or the fact that quarters have been dried off after a case of a case of mastitis [11]. Similarly, the higher prevalence of young cows with small udder shape is in accordance with expectations, as heifers and young cows normally have small udders. Previous studies have shown that parenchymal and alveolar cells in the udder grow until fifth lactation [16].

### Effect of lactation stage and parity in combination

The combined effect of lactation stage on the prevalence of asymmetric udders rises for younger cows but falls for older cows. This may partly be due to the fact, as discussed above, that at the start of lactation, udder tissue will be more voluminous and therefore the difference between the glands will be more pronounced. However, the reason for the decrease seen over the course of the lactation in the prevalence of older cows with asymmetric udders is most likely the fact that, farmers cull old cows with atrophy of a gland. This gives the misleading impression that the number of cows with atrophy is falling amongst the older cows (selection bias). The same could be the case when explaining the higher prevalence of older cows with deep udders, since they are the ones left in the herd. It is known that cows with deep udders have an increased risk of mastitis [17] as these udders have an increased tendency to become soiled and hence populated by bacteria. Therefore the reason for the observed falling prevalence of cows with deep udders over the course of the lactation is likely to be that the farmers are culling the old cows with deep udders.

The only teat shape, which was influenced by both lactation stage and parity, is short teat shape. This is very plausible, as one must expect some effect of the milking machine action on the teat [11]. Similarly, the degree of teat end callus may be affected by the milking machine, and therefore a gradual rise is seen during the lactation. These results correspond to the study done by Neijenhuis [10], and although there is some observer difference, all of the observers see the same tendency.

### Differences between farms

Variability in results between farms may reflect different conditions for doing observations, rather than true differences in the states of certain conditions in the cows. The presence of mange may serve as an example of this. When making the observations in the milking parlour, some farms had a big shield behind each cow, to protect the milker from kicks and manure in case this was relevant. This big shield made it difficult to observe for mange, and thus gave the possibly inaccurate result that the mange status differed significantly between the participating farms.

The prevalence of wounds, teat scarring and hardness of teats were also all found to be affected by the farm. Qualitative interviews with farmers [2] reveal that there are major differences in how farmers make their decisions regarding treatment of injuries and disease. For example, one farmer could have a policy where all cows with teat injuries should be culled as soon as possible, whereas another may not consider teat injuries to be a culling reason. These measurements could therefore be said to be factors, which may be directly influenced by herd management.

### Differences between animals

Soiled legs, hock lesions, udder consistency, teat skin, and teat end callus are all highly influenced by the effect of animal. All these variables are linked to the direct reactions of the animal to the environment. For example teat end callus has been found by Neijenhuis [12] to vary over the course of lactation as the teat canal mechanism adjusts the milking action in a very similar manner demonstrated in the results of this study.

In conclusion, there seem to be agreement between biologically plausible causes and the significance level of the individual effects (lactation stage, parity, farm and animal). This suggests that the variables may contribute an 'objective' view of the health status on the individual farm. Although there was overall general agreement amongst observers that the observations were easy to perform some of the variables may need a significantly improved training and description, e.g. photo guides, in order to be consistent between observers.

Generally, the results are consistent and biologically sound. The observed changes following lactation stage, parity or both do point to the relevance of the variables in a clinical examination and point to the fact that judgements of what is 'normal' and what is 'healthy' need to be viewed with a certain flexibility and in a context of farm, animal, lactation stage and parity.

Discussions based on this type of information, which cannot be obtained in any other manner, form an ideal 'meeting place' for farmer and veterinarian for making decision plans and strategies for changing health problems.
